# Assessment of Groundwater Quality Using Water Quality Index from Selected Springs in Manga Subcounty, Nyamira County, Kenya

**DOI:** 10.1155/2022/3498394

**Published:** 2022-03-14

**Authors:** Alice Makonjo Wekesa, Calford Otieno

**Affiliations:** Kisii University, Kisii, Kenya

## Abstract

We present the results of groundwater quality assessment that was done during the rainy season in November 2018 in the Manga region of Nyamira County, Kenya. Water samples were collected from three springs, Kiangoso, Kerongo, and Tetema, for the assessment. Water quality index was calculated based on pH, turbidity, nitrate, phosphate, calcium, magnesium, chloride, sulphates, fluoride, iron, total phosphorous, total hardness, total alkalinity, total dissolved solids, and total coliform. These fifteen parameters were analyzed and characterized according to standard methods and with reference to the World Health Organization and Kenya Bureau of Standards for physiochemical and bacteriological parameters which were then used in the calculation of water quality index. The water quality index was 21.32 for Kiangoso, 29.66 for Kerongo, and 25.64 for Tetema. The water quality index was found to be of excellent quality status at Kiangoso, while of good quality status at Kerongo and Tetema. The water quality index of Manga groundwater represented by the three springs therefore is less than 30 and can be used for drinking, irrigation, and industrial purpose. The present results are crucial for future management of groundwater in the Manga region.

## 1. Introduction

Water is essential for human survival hence termed as water is life [1, 2]. From the hydrological cycle, the availability of water both in the atmosphere, oceans, seas, lakes, and ground water forms a large percentage of the earth's composition [3]. However, not all water available either in all fore mentioned places may be suitable for human consumption as well as human activities like irrigation of crops, fish farming, or even all agricultural activities in totality because of water pollution [4–6]. Across the world today, water pollution is a major cause of undesirable water quality. This may be due to contamination of the water bodies by either emission of acidic gases into the atmosphere or as the release of waste industrial products into water bodies [7–12]. The use of pesticides and agricultural fertilizers which are categorized as anthropogenic factors [13, 14] to boost the growth of plants may also result into pollution and contamination of ground water which has been widely used as the main source of freshwater. All the anthropogenic activities mentioned are a result of increase in population over years [2] and unpredictable climatic changes [15–17].

It is therefore necessary that water quality investigation or assessment can be done to find out whether the available water from the termed reliable sources is safe for drinking [18, 19] and other uses. Water quality index which was originally designed by Horton in 1965 [20] and Brown [21] and later advanced on [22–27] is therefore a crucial tool when water quality assessments are being done [28–30] by many researchers across the world [31–34]. This entails the use of various physical, chemical, and biological parameters that are characterized and compared to a standard regulatory value which is then quality rated to obtain a single quality indicator termed water quality index [35–37]. The single value is important to any manager who needs precise and concise information on water quality.

## 2. Materials and Methods

### 2.1. Study Area

**Figure 1:**
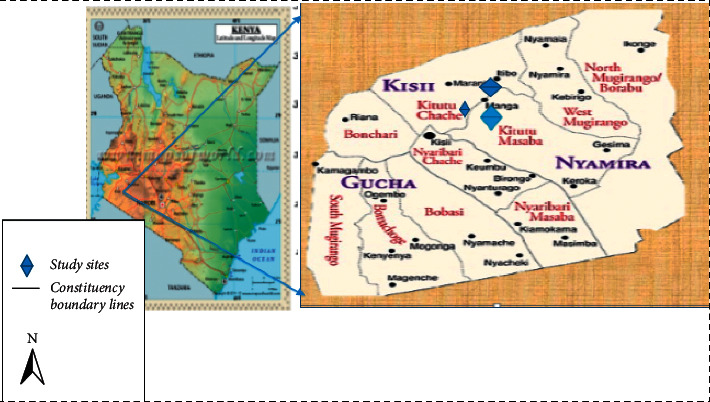
Map of Kenya showing the location of the Manga subcounty.

The study was conducted in Manga area (Figure 1). The area is found in the Western Kenya at the border of Nyamira and Kisii counties. It is a source of not only perennial and seasonal springs but also streams, for example, Kiogo, Nyabinyinyi, Nyatieko, Kiangoso, and Getwanyansi, which serve the people in two counties.

The area is also an agricultural zone with different agricultural activities and farming methods being practiced. The use of various pesticides and fertilizers is also being encouraged to boost the agricultural produce for the growing population as well as mitigation on unpredictable climatic changes.

Geologically, the area consists of granitic intrusions, conglomerates, and quartzite that vary in distribution across the area. For instance, SP1 which was Kiangoso spring is located in a rocky, quartzite region near the Manga ridge and seasonal in nature. SP2 which is Kerongo is a perennial spring, which is located in a less rocky area, while SP3 is Tetema spring that is also perennial and located in a basalt zone. Kiangoso is located at 000° 38′42.5″S and 034° 48′45.0″E and an elevation of 1785 m. Kerongo is located at 000° 39′ 03.7″S, 034° 49′ 08.7″E and elevation of 1844 m, while Tetema is located at 00° 39′ 08.4″S, 034° 48′ 55.4″E and at an elevation of 1823 m. Furthermore, Tetema spring is 435 m east from Kerongo spring and 866 m north from Kiangoso spring.

This region is selected because it is the main source of water for domestic use by the residents who practice agriculture by using pesticides and fertilizers.

### 2.2. Sample Collection

The samples were collected between 1.00 p.m. and 4.00 p.m. (East African Standard Time) from three springs during the rainy season of November 2018. These springs were identified since they were points of discharge of groundwater in the area and also prime sources of water for domestic use. This was done by letting water to flow freely from the improved spring into the 1.0 litre capacity plastic bottle that had been washed with distilled water and rinsed twice with water from the respective sampling spring. The water filled bottle was then immediately labelled to avoid confusion.

### 2.3. Sample Analysis

#### 2.3.1. On-Site Analysis

The on-site analysis was done using the Hanna combo H198129 waterproof tester for nonconservable parameters that were of importance in the calculation of water quality index in the study. These included the pH and total dissolved solids (TDS). The turbidity was also determined in situ by the nephelometric method.

#### 2.3.2. Laboratory Analysis

The samples were taken to the laboratory in Kisumu in which twelve parameters such as nitrates, phosphate, calcium, magnesium, chlorides, sulphates, fluorides, iron, total phosphorous, total hardness, total alkalinity, and total coliforms were characterized in accordance to the standard methods for physiochemical and bacteriological parameters as prescribed by APHA [38] which gives standard methods for examination of water and wastewater and with reference to the World Health Organization (WHO) standards [39] and Kenya Bureau of Standards (KEBS) [40] standards. For instance, phosphate, nitrate, iron, fluorides, and total hardness were determined using the spectrophotometric method. The absorbance in the fluoride determination is being set at 570 nm. Magnesium, calcium, chloride, and sulphate were determined using the titration method.

### 2.4. Calculation of Water Quality Index

The water quality index calculation procedure was a modification of the previous procedures used by Bouslah et al. [2], Chatterji and Raziuddin [35], Brown et al. [36], Asadollahfardi [37], and Tripaty and Sahu [41] in their research studies. In this study, each parameter was assigned a unit weight (Wu) in the scale of 1–5, in which 1 represents the least health effect and 5 represents the adverse health effect the parameter causes when present in drinking water. This unit weight (Wu) of the parameter was then used to calculate the relative weight (Wr). This was done by finding the quotient of the specific unit parameter and the sum of unit weights as shown by the following equation:

(1) $$\begin{array}{c} Wr=\frac{Wu}{\sum Wu}. \end{array}$$

The quality rating *Q*_*r*_ of each parameter was obtained using the following equation:

(2) $$\begin{array}{c} Q_{r}=100\left[\frac{Vn\;-V_{1}}{V_{S\;-\;V_{1}}}\right], \end{array}$$

where *V*_*n*_ is the observed value, *V*_*s*_ is the WHO/KEBS limit, and *V*_1_ is the ideal value.

For drinking water, the ideal value for the parameters used in this study is said to be zero except for pH which is 7.0 [2, 41].

The relative weight and respective quality rating of a parameter are multiplied to give the parameter subindex (PI_S_) value as shown in the following equation:

(3) $$\begin{array}{c} \text{PIs}=WrQr. \end{array}$$

The sum of parameter subindices gives the water quality index (WQ1) as shown in the following equation:

(4) $$\begin{array}{c} WQI=\sum \text{PIs}. \end{array}$$

The water quality index (WQI) was finally compared to the water quality status (WQS) as given in Table 1.

## 3. Result and Discussion

### 3.1. Physiochemical Parameters

The results of physical, chemical, and biological parameters assessed in the study and their respective recommended standards are given in Table 2.

From Table 2, it is clear that all parameters except pH are within the maximum acceptable limits required by the WHO and KEBS.

A descriptive statistical analysis obtained using Sigma Plot 12.5 version of groundwater parameters of the Manga region is as given in Table 3.

#### 3.1.1. pH

The pH values obtained are 5.7, 5.1, and 5.4 for SP1, SP2, and SP3, respectively. These values are lower than the accepted values by either the WHO or KEBS. The lower values of pH were also obtained by Wanyoike et al. [42] in their research on the bubbling springs of Kilibwoni in Nandi County in Kenya. The values obtained from spring were 4.78 and 4.93 in rain season and dry season, respectively. Furthermore, all the pH values obtained in their study are considered acidic and attributed to geological underground activities. In the case of the Manga region, the pH is slightly acidic which in this case represents the oligotrophic nature of spring that is supported by very low levels of plant supporting parameters associated with agricultural fertilizers such as chloride, nitrate, sulphate, orthophosphate, and total phosphate.

#### 3.1.2. Nitrate

Nitrate concentration levels are 1.2, 0.88, and 0.68 corresponding to SP3, SP2, and SP1, respectively, which is within the acceptable limit of less than 10. These values are also comparable closer to values obtained for spring in Nandi County [42] that had nitrate value of 1.6 in the rain season. According to [43], water from three springs is normal and safe for use.

#### 3.1.3. TDS

TDS values of water from three springs are less than 25 mg/l and hence categorized by Freeze and Cherry [44] as fresh water since these TDS values are less than 100 mg/l. In addition, these TDS values fall in the category of excellent palatability of drinking water as per [45] because this value is less than 300 mg/l. TDS which is also discussed by Wanyoike et al. [42] as clarity and cleanness of water in their research was found to be 28 in the rainy season.

#### 3.1.4. Turbidity

Turbidity values of this region ranges between 0.84 N.T.U and 1.05 N.T.U and having a mean of 0.93 N.T.U, which is within the acceptable maximum limit of 5 N.T.U by the WHO and KEBS. A comparison with turbidity level of 4.7 in the rainy season from [42] show that spring water from the Manga region during that period had a higher clarity level and minimal suspended material.

#### 3.1.5. Fluoride, Iron, and Coliforms

The iron, fluoride, and coliform levels were not detected in the water samples; hence, the significance value is zero.

#### 3.1.6. Total Hardness and Total Alkalinity

The total hardness which results from the presence of alkaline earth metals ranges from 8 mgCaCO_3_/l to 10 mgCaCO_3_/l with a mean value of 9.33 mgCaCO_3_/l. This according to Sawyer [46] is an association of soft water since this level is less than 75 mgCaCO_3_/l.

The total alkalinity which results from the presence of alkaline earth metals ranges from 17 mgCaCO_3_/l to 20 mgCaCO_3_/l.

#### 3.1.7. Calcium and Magnesium

The levels of calcium and magnesium of water from three springs is very low and within the limits of maximum acceptable by KEBS of 150 mg/l and 100 mg/l, respectively.

#### 3.1.8. Chlorides and Sulphates

The level of chloride at SP1, SP2, and SP3 is 3 mg/l, 2 mg/l, and 1 mg/l, respectively, while the level of sulphate is 4 mg/l, 2 mg/l, and 2 mg/l for SP1, SP2, and SP3, respectively. Moreover, the level of chloride and sulphate is higher at SP1 than at SP2 and SP3. The level of sulphate at SP1 is twice that of SP2 and SP3, while the level of chloride at SP3 is lower than the levels at SP1 and SP2. All the levels of chloride and sulphate from the three selected springs from the region is within is within the acceptable limit of 250 mg/l and 400 mg/l, respectively. However, the levels of chloride and sulphate are higher than those obtained for the bubbling spring in Nandi County [42] of 0.499 and 0, respectively.

#### 3.1.9. Phosphates and Total Phosphorous

The level of phosphate from the Manga region ranges between 0.08 and 0.24. These values are lower than the values obtained by Wanyoike et al. [42] that ranged between 0.74 and 0.90 for spring in rain and dry seasons, respectively. However, a comparison with values obtained from Malaget in Kenya [47] of 0.002–0.037 shows that the phosphate level in Manga region is more than that of Malaget but less than that of Kilibwoni, but within the accepted limit by KEBS of 30 mg/l.

The obtained values of phosphorus ranges between 0.16 mg/l and 0.36 mg/l, hence, within the acceptable limit less than 2 by the WHO.

The level of orthophosphate and total phosphorous as very low and in both categories is less than 1 mg/l.

### 3.2. Water Quality Index

Table 4 provides the unit weight, relative weight, quality rating, and sub-index values that were obtained and used to calculate water quality index.

Using the fifteen parameters given in Table 3, the water quality index was 21.32 for SP1, 29.66 for SP2, and 25.64 for SP3. The water quality index at SP2 is higher than that at SP1 and SP3. However, the water quality index ranges from 21.32 to 29.66. This is majorly due to the low pH level values in the Manga area that primarily cause a metallic taste of water. The water quality index of the Manga groundwater represented by the three springs therefore is less than 30. The water with such a water quality index from Table 1 can be used for drinking, irrigation, and industrial purpose.

Basing on the water quality categories as discussed by [35–37] given in Table 1, SP1 which is Kiangoso spring has an excellent water quality status, while SP2 and SP3 is Kerongo and Tetema, respectively; the water quality status is good. Furthermore, the water quality index and water quality status obtained for the respective sources are given in Table 5.

In Kenya, similar results in the two categories were obtained by Kirui [47] in Malaget in Kericho in water samples from S1 and S2 being excellent quality while from S3 and S4 had good quality. In addition, a study by Achieng et al. [48] on Nyando River in Muhoroni in Kenya showed higher values of water quality index than this of Manga region. This study by Achieng et al. had water quality index values ranging from 51.88 to 101.131 and depicted that water at Homalime and Wasao was of poor water quality and unsuitable for drinking at Kipchui.

An assessment done on groundwater by Ibrahim [49] in Jordan showed a wide range of water quality index than that of Manga region and ranging from 40 to 4295. In the assessment, the poor and unsuitable status of the water quality was attributed to anthropogenic activities occurring around the area of study. Moreover, higher values of water quality index were also obtained in Algeria [2] at Koudiat Medouar reservoir that ranged from 99.08 to 174.72.

## 4. Conclusion

Manga groundwater represented by three springs is therefore said to have water quality index of less than 30, ranging between good and excellent in quality, soft, safe for use by both people and livestock, and excellent palatability of drinking, irrigation, as well as industrial purpose.

With the current state of unpredictable climatic change and population growth, these results presently obtained from the water assessment shall be useful in future for management of the Manga groundwater.

## Figures and Tables

**Table 1:** Water quality index and water quality status as adapted from [35–37].

WQI	WQS	Possible use
0 ≤ 25	Excellent	Drinking, irrigation, and industrial use
26 ≤ 50	Good	Drinking, irrigation, and industrial use
51 ≤ 75	Poor	Irrigation and industrial use
76 ≤ 100	Very poor	Irrigation
˃100	Unsuitable for drinking	Requires proper treatment before use

**Table 2:** Parameter characterization, recommended standards, and agency.

Parameter	Unit	SP1	SP2	SP3	Standard error	Standard	Reference agency [39, 40]
pH	pH scale	5.7	5.1	5.4	0.173	6.5–8.5	WHO/KEBS
Turbidity	N-T-U	0.9	0.84	1.05	0.0624	Max 5	WHO/KEBS
Nitrate	mgNO_3_/l	0.68	0.88	1.2	0.151	Max 10	WHO/KEBS
Phosphate	mg/l	0.14	0.24	0.08	0.0467	Max 30	KEBS
Calcium	mg/l	8	6	9	0.882	Max 150	KEBS
Magnesium	mg/l	1.2	0.9	0.67	0.153	Max 100	WHO/KEBS
Chloride	mg/l	3	2	1	0.577	Max 250	WHO/KEBS
Fluoride	mg/l	0	0	0	0.000	Max 1.5	WHO/KEBS
Sulphate	mg/l	4	2	2	0.667	Max 400	KEBS
Iron	mg/l	0	0	0	0.000	Max 0.3	WHO/KEBS
Total phosphorous	mg/l	0.28	0.36	0.16	0.0581	Max 2	WHO
Total hardness	mgCaCO_3_/l	10	8	10	0.667	Max 300	KEBS
Total alkalinity	mgCaCO_3_/l	20	17	20	1.000	Max 500	WHO
TDS	mg/l	25	22	23	0.882	Max 1000	KEBS
Coliforms	Colonies/100 ml	0	0	0	0.000	0	WHO/KEBS

**Table 3:** Statistical analysis of the observed groundwater quality parameters in the Manga region.

Parameter	Min	Max	Range	Mean	Std. deviation	C.I of mean	KS distance	KS prob	SWilk W	SWilk prob
pH	5.1	5.7	0.60	5.4	0.30	0.745	0.175	0.654	1.00	1.00
Turbidity	0.84	1.05	0.21	0.93	0.0624	0.269	0.276	0.404	0.942	0.537
Nitrate	0.68	1.20	0.52	0.92	0.262	0.652	0.227	0.569	0.983	0.747
Phosphate	0.08	0.24	0.16	0.153	0.808	0.201	0.232	0.555	0.980	0.726
Calcium	6.00	9.00	3.00	7.667	0.882	3.795	0.253	0.487	0.964	0.637
Magnesium	0.67	1.20	0.53	0.923	0.153	0.660	0.202	0.627	0.994	0.855
Chloride	1.00	3.00	2.00	2.00	1.00	2.484	0.175	0.654	1.000	1.000
Fluoride	0.00	0.00	0.00	0.000	0.000	0.000	0.000	0.000	0.000	<0.001
Sulphate	2.00	4.00	2.00	2.667	1.155	2.868	0.385	0.089	0.750	<0.001
Iron	0.00	0.00	0.00	0.000	0.000	0.000	0.000	0.000	0.000	<0.001
Total phosphorous	0.16	0.36	0.20	0.267	0.101	0.250	0.219	0.590	0.987	0.780
Total hardness	8.00	10.0	2.00	9.333	1.155	2.868	0.385	0.089	0.750	<0.001
Total alkalinity	17.0	20.0	3.00	19.00	1.000	4.303	0.385	0.089	0.750	<0.001
TDS	22.0	25.0	3.00	23.33	1.528	3.795	0.253	0.487	0.964	0.637
Coliforms	0.00	0.00	0.00	0.000	0.000	0.000	0.000	0.000	0.000	<0.001

**Table 4:** Unit weight, relative weight, quality rating, and subindex values.

Parameter	Weight		SP1		SP2		SP3	
	*W*_*u*_	*W*_*r*_	*Q*_*r*_	PI_s_	*Q*_*r*_	PI_s_	*Q*_*r*_	PI_s_
pH	4	0.06897	260	17.9322	380	26.2086	320	22.0704
Turbidity	3	0.05172	18	0.93096	16.8	0.86890	21	1.08612
Nitrate	5	0.08621	6.8	0.58623	8.8	0.75865	12	1.03452
Phosphate	3	0.05172	0.035	0.00181	0.06	0.00310	0.02	0.00103
Calcium	3	0.05172	5.3	0.27412	4	0.20688	6	0.31032
Magnesium	3	0.05172	1.2	0.06206	0.9	0.04655	0.67	0.03465
Chloride	5	0.08621	1.2	0.10345	0.8	0.06897	0.4	0.03448
Fluoride	5	0.08621	0	0	0	0	0	0
Sulphate	5	0.08621	1	0.08621	0.5	0.04311	0.5	0.04311
Iron	3	0.05172	0	0	0	0	0	0
Total phosphorous	3	0.05172	14	0.72408	18	0.93096	8	0.41376
Total hardness	3	0.05172	3.33	0.17223	2.67	0.13809	3.33	0.17223
Total alkalinity	4	0.06897	4	0.27588	3.4	0.23450	4	0.27588
TDS	4	0.06897	2.5	0.17243	2.2	0.15173	2.3	0.15863
Coliforms	5	0.08621	0	0	0	0	0	0
WQI				21.32		29.66		25.64

**Table 5:** Water quality index and water quality status.

Water source	WQI	WQS
SP1	21.32	Excellent
SP2	29.66	Good
SP3	25.64	Good

## Data Availability

The data used to support the findings of this study are available from the corresponding author upon request.
